# Gadolinium retention in a rat model of subtotal renal failure: are there differences among macrocyclic GBCAs?

**DOI:** 10.1186/s41747-023-00324-1

**Published:** 2023-03-01

**Authors:** Roberta Bonafè, Alessandra Coppo, Roberta Queliti, Simona Bussi, Federico Maisano, Miles A. Kirchin, Fabio Tedoldi

**Affiliations:** 1 Bracco Imaging SpA, Bracco Research Centre, Via Ribes 5, 10010, Colleretto Giacosa, TO Italy; 2 Bracco Imaging SpA, Global Medical & Regulatory Affairs, Milan, Italy

**Keywords:** Gadobutrol, Gadoteridol, Gadoterate meglumine, Gadolinium, Renal insufficiency

## Abstract

**Background:**

Gd levels are higher in tissues of animals with compromised renal function, but studies to compare levels after exposure to different macrocyclic gadolinium-based contrast agents (GBCAs) are lacking. We compared Gd levels in tissues of subtotally nephrectomised (SN) rats after repeated exposure to macrocyclic GBCAs.

**Methods:**

Sprague–Dawley SN male rats (19 per group) received 16 injections of gadoteridol, gadobutrol, or gadoterate meglumine at 0.6 mmol Gd/kg 4 times/weeks over 4 weeks. A control group of healthy male rats (*n* = 10) received gadoteridol at the same dosage. Plasma urea and creatinine levels were monitored. Blood, cerebrum, cerebellum, liver, femur, kidney(s), skin and peripheral nerves were harvested for Gd determination by inductively coupled plasma-mass spectrometry at 28 and 56 days after the end of treatment.

**Results:**

Plasma urea and creatinine levels were roughly twofold higher in SN rats than in healthy rats at all timepoints. At day 28, Gd levels in the peripheral nerves of gadobutrol- or gadoterate-treated SN animals were 5.4 or 7.2 times higher than in gadoteridol-treated animals (*p* < 0.001). Higher Gd levels after administration of gadobutrol or gadoterate *versus* gadoteridol were also determined in kidneys (*p* ≤ 0.002), cerebrum (*p* ≤ 0.001), cerebellum (*p* ≤ 0.003), skin (*p* ≥ 0.244), liver (*p* ≥ 0.053), and femur (*p* ≥ 0.271). At day 56, lower Gd levels were determined both in SN and healthy rats for all GBCAs and tissues, except the femur.

**Conclusions:**

Gd tissue levels were lower following gadoteridol exposure than following gadobutrol or gadoterate exposure.

## Key points

• In subtotally nephrectomised rats, lower Gd tissue levels were observed after gadoteridol than after gadobutrol or gadoterate.
• Lower Gd levels after gadoteridol were particularly evident in the brain, peripheral nerves, and kidneys.
• Tissue Gd elimination over time occurs for all three contrast agents but was less evident in the femur.

## Background

Gadolinium-based contrast agents (GBCAs) have been available for clinical use since 1988 and have generally been considered to have a very good safety profile in terms of immediate, acute-type adverse reactions [1, 2]. However, a report in 2006 [3] of an association between the administration of certain GBCAs and a potentially fatal disease subsequently termed nephrogenic systemic fibrosis (NSF) in patients with severe renal insufficiency (estimated glomerular filtration rate < 30 mL/min/1.73 m^2^) led to markedly increased scrutiny of the safety of these agents.

Whereas contraindication to linear GBCAs in patients with severe renal insufficiency effectively eliminated NSF as a risk associated with GBCA use, a report in 2014 [4] of increased signal intensity at magnetic resonance imaging in specific brain structures (primarily the dentate nucleus and globus pallidus) on unenhanced T1-weighted images of patients that had previously undergone multiple GBCA intravenous injections once again raised concern about GBCA safety. Subsequent demonstration of retained gadolinium (Gd) in brain tissue samples from decedents undergoing autopsy [5] and numerous demonstrations from studies in animals of retained Gd in brain and body tissues following multiple GBCA exposures [6–18] have confirmed that Gd is retained to a greater or lesser extent after administration of all GBCAs but that the greatest levels of retained Gd are seen after administration of linear nonionic GBCAs, *i.e.*, those with a clearer association with NSF. A consequence of NSF and the demonstration of greater Gd retention after linear GBCA administration has been a move away from linear GBCAs towards a prevalent use of macrocyclic GBCAs for extrahepatic clinical applications. Among the macrocyclic GBCAs, gadoteridol (ProHance®) has been shown to result in lower levels of retained Gd compared to both gadobutrol (Gadovist®/Gadavist®) and gadoterate meglumine (Dotarem®/Clariscan®) in rats with normal renal function [15–18].

Given that severe renal impairment resulting in reduced renal elimination and prolonged elevated blood GBCA levels is considered a primary factor leading to NSF in humans given certain linear GBCAs, several studies have looked at the impact of subtotal renal failure on Gd retention and/or T1 signal hyperintensity in deep cerebellar nuclei in animals [19–21]. These studies have shown that renal impairment potentiates Gd retention in deep cerebellar nuclei and that greater Gd retention and T1 signal hyperintensity are seen with linear GBCAs. As yet, however, no studies have compared Gd retention after exposure to different macrocyclic GBCAs in animal models of subtotal renal failure.

This comparative study was performed in rats with subtotal renal failure to determine Gd levels in various organs and tissues following cumulative administration of the three macrocyclic GBCAs currently available in Europe: gadoteridol, gadobutrol, and gadoterate meglumine.

## Methods

The study was performed at Bracco Imaging SpA (Italy), according to site-specific procedures established by the relevant Quality Assurance Unit. Procedures were conducted according to national and international regulations (L.D. 26/2014; Directive 2010/63/EU) under authorisation no. 1229/2020-PR, released on December 31, 2020, by the “Direzione Generale della Sanità Animale e dei Farmaci Veterinari, Ufficio VI” from the Italian Ministry of Health.

### Animal model and administration protocol

Fifty-seven subtotally nephrectomised (SN) Sprague Dawley Caesarean-derived male rats as per International Genetic Standardization were supplied by Charles River Laboratories (France). Each rat had undergone an initial operation at six weeks of age to remove the upper and lower poles of one kidney and then a second operation one week later to excise the remaining kidney, thereby achieving five sixths nephrectomy. The animals were randomised to treatment groups after one week of recovery and an additional eight days of acclimatisation. At the start of dosing, all SN rats were aged between 9 and 10 weeks and weighed between 288 and 387 g. An additional 10 non-SN Sprague Dawley Caesarean-derived male rats (Charles River Laboratories, Italy) aged between 9 and 10 weeks and weighing between 347 and 404 g at the start of dosing were utilised as a control group. Only male rats were used because gender was not expected to impact Gd retention [22].

Three GBCAs were compared: gadoteridol (ProHance; Bracco Imaging SpA, Milan, Italy; batch numbers V1904 and V19732, expiry: 02/2022 and 06/2022, respectively), gadobutrol (Gadovist; Bayer, Leverkusen, Germany; batch numbers KT02930 and KT05S2C, expiry: 10/2021 and 10/2022, respectively), and gadoterate meglumine (Dotarem; Guerbet LLC, Villepinte, France; batch number 20GD001A02, expiry: 12/2022).

Animals were housed under controlled conditions at a temperature of 22 °C ± 2 °C, humidity of 55% ± 10%, and 12-h light/dark cycles. Food pellets and filtered water from municipal services were provided ad libitum. After eight days of acclimatisation, SN animals were randomly assigned to one of three exposure groups (19 rats per group): group 1 (gadoteridol 0.5 mol/L), group 2 (gadobutrol 1 mol/L), and group 3 (gadoterate meglumine 0.5 mol/L); ten healthy animals were assigned to group 4 (gadoteridol 0.5 mol/L).

All rats received intravenous injections of GBCA at a dose of 0.6 mmol/kg 4 times a week, for 4 consecutive weeks (16 administrations overall), for a total cumulative dose of 9.6 mmol/kg. This daily dose corresponds to a human clinical dose of 0.1 mmol/kg based on the extrapolation factor for rats described in the US Food & Drug Administration guidance for human equivalent dose [23]. GBCA administration was performed at room temperature into the lateral vein of the tail, at 2 mL/min using a Harvard infusion pump (Holliston, MA, USA), through a 5-mL Terumo syringe connected to a 25-G butterfly needle.

**Fig. 1:**
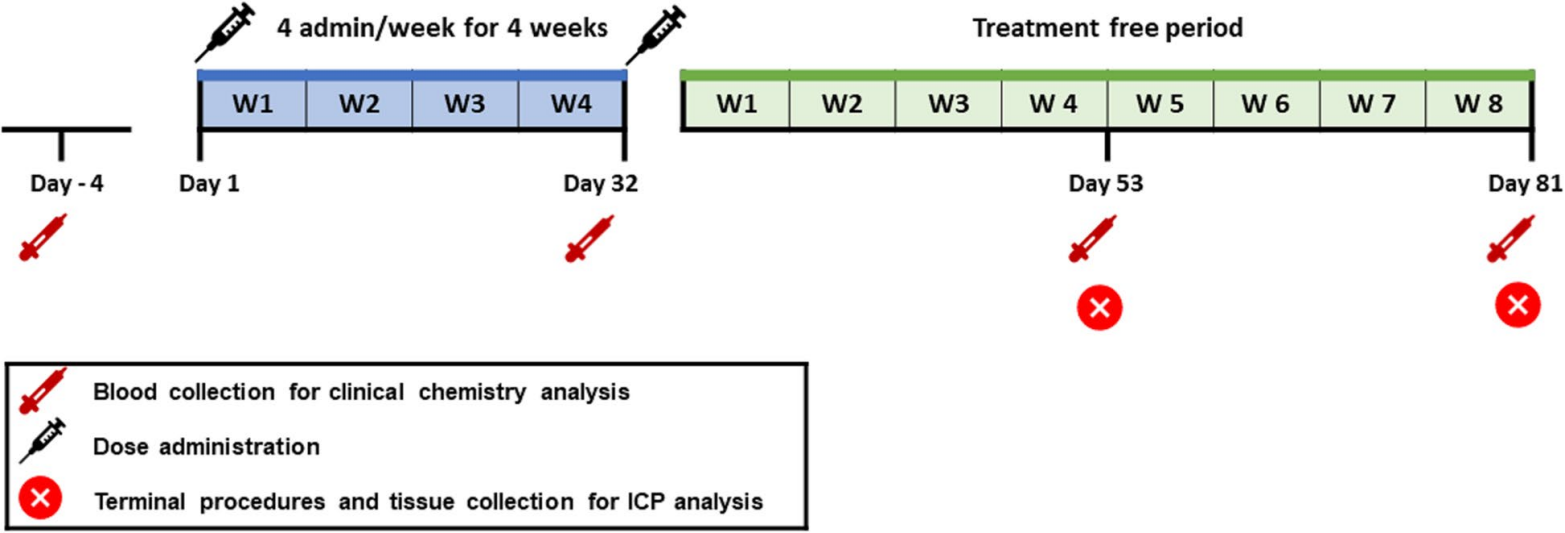
Experimental design. Subtotally nephrectomised and healthy rats were randomised to 4 exposure groups for each scheduled sacrifice at day 28 (*n* = 10 rats in groups 1, 2, and 3; *n* = 5 in group 4) or day 56 (*n* = 9 in groups 1, 2, and 3; *n* = 5 in group 4) after the end of the 4-week treatment period. Animals in each group received a single daily intravenous injection of the respective gadolinium-based contrast agent at a dose of 0.6 mmol/kg body weight 4 times a week for 4 consecutive weeks. At scheduled sacrifice, blood and tissues were harvested for Gd determination by inductively coupled plasma/mass spectrometry. As not all the animals completed the study, the sample size was reduced to 8 rats in groups 1 and 2, to 9 in group 3 at day 28, and to 8 in groups 1 and 3, and 7 in group 2 at day 56. Blood was sampled for urea and creatinine determinations predose (day − 4), at the end of dosing (day 32) and before scheduled sacrifice (day 53 and day 81)

Animals from each group were sacrificed after washout (treatment-free) periods of 28 days (8 rats of groups 1 and 2, 9 rats of group 3, and 5 rats of group 4) or 56 days (8 rats of groups 1 and 3, 7 rats of group 2, and 5 rats of group 4) from the end of the 4-week treatment period (Fig. 1).

### Observations

During the treatment period, all animals were inspected before and after dosing for any clinical signs or reactions to treatment. During the treatment-free period, all animals were inspected once daily. A full clinical examination was performed pretest and then weekly during the treatment and treatment-free periods.

### Laboratory investigations

Blood for clinical chemistry tests (approximately 0.4 mL) was collected in lithium heparin tubes from the lateral tail vein at day -4 (predosing) and day 32 (postdosing). At the last collection time point (at sacrifice at day 53 and day 81), blood was sampled from the abdominal aorta at sacrifice (at day 28 or day 56 after treatment). Urea and creatinine were analysed in plasma samples, obtained after centrifugation (2,100 g for 10 min), and frozen at -20 °C. A ILab 300 Plus analyser (Werfen, Milan, Italy) was used.

### Pathology

Animals were anaesthetised with SevoFlo at an induction rate of 3 to 4% and subsequently by intramuscular injection of 0.20 mL/kg of Zoletil 50/50 (20 mg/kg) and 0.25 mL/kg of Rompun (5 mg/kg). Anaesthetic doses were calculated based on the last recorded body weight. Animals were euthanised and bled after collection of blood from the abdominal aorta. Blood was collected in tubes containing lithium heparin gel and stored at 2 to 8 °C until sample preparation was required for clinical chemistry or inductively coupled plasma/mass spectrometry (ICP-MS) analyses. After exsanguination, a complete macroscopic postmortem examination was performed. Any abnormal findings were recorded. Thereafter, each animal was dissected to obtain the following tissues for ICP-MS determination of Gd: cerebrum, cerebellum, liver, kidneys, peripheral nerves (lumbosacral plexus, sciatic, and tibial nerves from both legs), femur, and skin. Tissues were sampled from all animals using sterile disposable surgical instruments to avoid cross-contamination, weighed, then placed in disposable tubes and frozen at -80 °C ± 10 °C. A total of 461 blood/tissue samples were collected (58 animals; 7 tissue samples [− 3 missing skin samples], and 1 blood sample per animal).

### Determination of total Gd

Sample collection and digestion for the destruction of the organic matrix were performed at the Test Facility (Bracco Imaging SpA; Colleretto Giacosa, Turin, Italy) by subjecting each biological matrix to different mineralisation processes using a Digiprep system (SCP Science, Baie D'Urfé, Canada). Blood samples (0.25 mL) were mixed with 4 mL of concentrated nitric acid (67 to 69% w/w), 1 mL of hydrochloric acid (34% to 37% w/w), and 2 mL of 30% w/w hydrogen peroxide, then heated for 180 min at 120 °C in a DigiBlock (SCP Science). Cerebrum, cerebellum, and peripheral nerves (0.2 g to 2.0 g weight range); femur (0.8 g to 2.0 g weight range); and kidneys (0.15 to 1.5 g weight range for SN animals, 1.5 to 3.5 g weight range for healthy animals) were digested using a two-step procedure: samples were mixed with 10 mL of concentrated nitric acid, allowed to react for about 15 min at room temperature, then heated for 10 min at 120 °C in the DigiBlock, paused for 10 min, and then heated for an additional 100 min. All procedures were repeated after the addition of 5 mL of 30% hydrogen peroxide. Liver samples were homogenised in an Ultra-Turrax disperser (Ika, Staufen, Germany), adding 1 mL of purified water for every 3 g of the liver to facilitate homogenisation of the samples. The water/sample ratio was taken into account for the weighing step, in which about 6.7 g of the homogenised sample was prepared for digestion. Skin samples (0.5 to 0.6 g weight range) were mixed with 8 mL of concentrated nitric acid and 2 mL of hydrochloric acid and heated for 180 min at 130 °C in the DigiBlock. ICP-MS analysis of mineralised tissue samples was performed at LabAnalysis S.r.l (Casanova Lonati, Pavia, Italy). For this analysis, blood, cerebrum, cerebellum, peripheral nerves, liver, and kidney samples were brought to 20 mL with purified water; samples of skin were brought to 50 mL with purified water; samples of femur were brought to 20 mL with purified water then diluted 1:10; and 5 mL of this sample solution was brought to 50 mL with purified water. All samples were filtered through 0.45-μm filters before analysis. The quality control samples were prepared in nitric acid and water mixture from certified reference solutions (gadolinium reference solution, CPA Chem, Gd 1,000 mg/L or terbium reference solution, Tb 1,000 mg/L) for each analytical sequence (in duplicate) at three different concentrations (low, medium, and high). Quality control samples were analysed after every 20 test samples.

The lower limit of quantitation (LLOQ) for Gd was 0.05 nmol/mL for blood; 0.05 nmol/g for the cerebrum, cerebellum, and peripheral nerves; 0.1 nmol/g for liver; 0.17 nmol/g for kidney; 0.25 nmol/g for femur; and 0.5 nmol/g for skin.

### Statistical analysis

Gadolinium concentration was expressed as nmol Gd/g of wet tissue or nmol Gd/mL for blood. Statistical analysis was performed on blood chemistry and Gd content in organs, to compare groups 1, 2, and 3 *versus* group 4, and groups 1, 2, and 3 among each other.

The Dixon test [24] was used before formal data analysis to highlight possible anomalous data points. Data were analysed based on the distribution and homogeneity of variance among groups. If the data were normally distributed based on quantile–quantile plots and Shapiro–Wilk’s test [25], Levene’s test [26] was utilised to test the homogeneity of variance among groups. These tests were performed at the significance level of *α* = 0.05, whereas subsequent tests were performed at three different significance levels: *α* = 0.05, 0.01, and 0.001. To test the null hypothesis that treatment groups have the same distribution, the analysis of variance (ANOVA) test was applied in the case of homogeneous variances. The Welch test [27] was used if variances were not homogenous. If the results of the ANOVA or Welch tests were significant, pairwise multiple comparisons were performed with the method proposed by Tukey [28] in the case of homogeneous variances, and Dunnett’s T3 test [29] in the case of nonhomogeneous variances. If data were not normally distributed, the null hypothesis was tested by nonparametric Kruskal–Wallis one-way analysis of variance. Pairwise multiple comparisons were performed if significant differences between treatment groups were detected. Adjusted *p*-values were calculated for multiple comparisons with the Bonferroni correction. All analyses were performed using SPSS v. 27.0 (SPSS, Chicago, IL, USA).

## Results

### Clinical signs and body weight

Nine SN animals did not complete the study. Three rats treated with gadoteridol, four rats treated with gadobutrol, and two rats treated with gadoterate were humanely euthanised due to their poor clinical conditions during the treatment and/or recovery periods (from day 5 to day 30). Early deaths on day 5 were considered due to incomplete recovery from surgery, as evidenced by macroscopically appreciable gross lesions. Conversely, the incidence of subsequent deaths was similar to incidences observed in previous studies in which GBCAs were repeatedly administered to rodent models of renal failure [19, 20, 30]. These deaths were related to the repeated daily intravenous administrations required by the experimental protocol rather than to the administered GBCAs themselves. No treatment-related clinical signs nor changes in body weight were observed in the surviving animals. Body weights were slightly higher in healthy animals (group 4) than in SN animals (groups 1, 2, and 3), throughout the study. This finding was not considered treatment-related as it was already observed on day 1, before the initiation of treatments (3–7% lower body weights for animals in groups 1, 2, or 3 *versus* animals in group 4).

### Blood chemistry

**Fig. 2:**
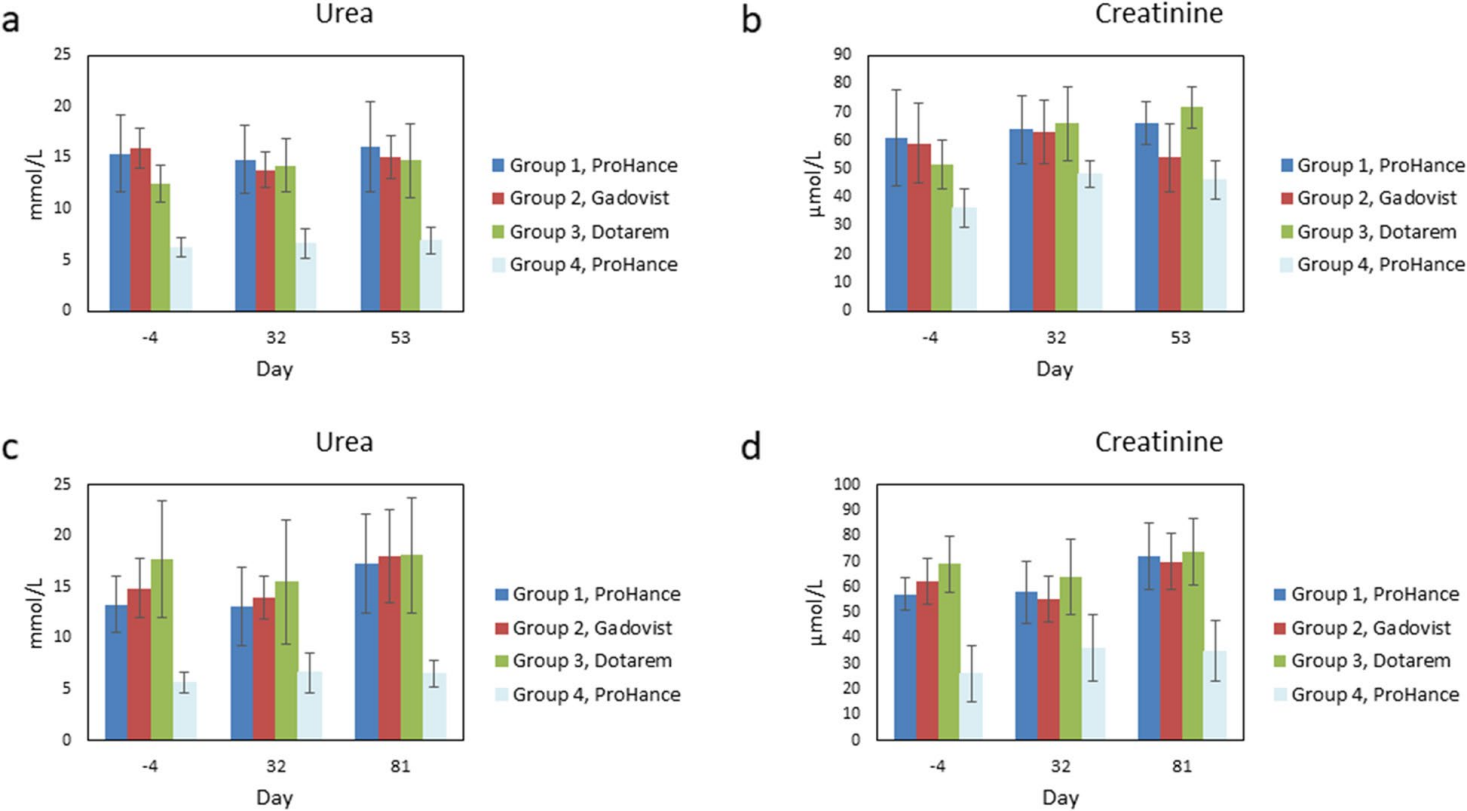
Plasma urea and creatinine levels. Urea and creatinine levels (mean ± standard deviation) determined before the start of gadolinium-based contrast agent administration (day -4), immediately after the end of dosing (day 32) and at scheduled sacrifices: 28 days post-treatment (day 53, **a**, **b**) or 56 days post-treatment (day 81, **c**, **d**). The error bars represent the standard deviation of measurements within the groups (group 1, gadoteridol, *n* = 8 in **a** to **d**; group 2, gadobutrol, *n* = 8 in **a** and **b**, *n* = 7 in **c** and **d**; group 3, gadoterate, *n* = 9 in **a** and **b**, *n* = 8 in **c** and **d**; group 4, gadoteridol, *n* = 5 in **a** to **d**)

The model of chronic kidney disease induced by subtotal nephrectomy was associated with increase in basal urea and creatinine plasma levels in all SN rats (groups 1, 2, and 3) compared with healthy rats (group 4, gadoteridol treated). Overall, urea and creatinine plasma levels in SN rats (groups 1, 2, and 3) were consistently about twofold higher than those in healthy rats (group 4, gadoteridol treated) before treatment (day -4, *p* ≤ 0.022 or *p* ≤ 0.010), at the end of treatment (day 32, *p* ≤ 0.048 or *p* ≤ 0.005) and at sacrifice (day 53, *p* ≤ 0.002; or day 81, *p* ≤ 0.011 or *p* ≤ 0.003). Over time, no treatment-related effects on urea and creatinine plasma levels were observed (Fig. 2). On day 53, significantly lower (*p* ≤ 0.002) creatinine levels were observed in animals treated with gadobutrol compared with those treated with gadoterate (Fig. 2b), but this difference was considered not biologically relevant as it was no longer present at day 81.

### Gadolinium content

**Fig. 3:**
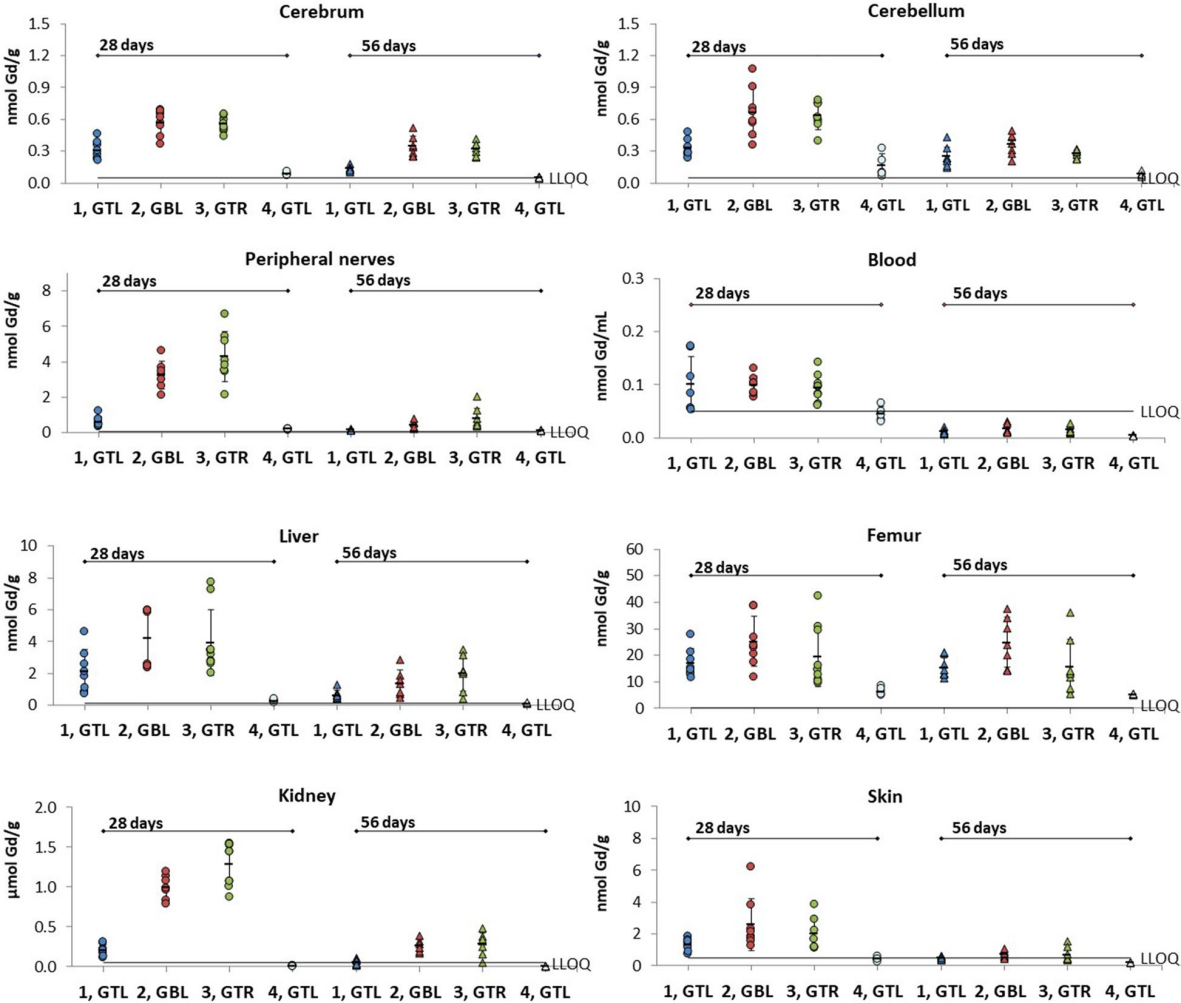
Gd content in different tissues. Gd content determined by inductively coupled plasma/mass spectrometry (individual values, mean ± standard deviation) in different tissues at day 28 and day 56 post-treatment. The error bars represent the standard deviation of measurements within the groups. On the x axis: group 1, gadoteridol (1, GTL), *n* = 7 or 8; group 2, gadobutrol (2, GBL), *n* = 6, 7, or 8; group 3, gadoterate (3, GTR), *n* = 7, 8, or 9; and group 4, (4, GTL), *n* = 4 or 5). On the y axis: nmol (µmol for kidney) Gd/g of wet tissue (or mL for blood)

The mean ± standard deviation (SD) Gd contents across groups and tissues are presented in Tables 1 and 2 and Fig. 3. A total of 461 tissue samples were analysed, each sample corresponding to an individual organ excised at necropsy. Among these 461 samples, data points from 18 (3.9%) samples were considered outliers based on the Dixon test. No more than a single outlier was identified in each sample group of animals and tissues. These data points were excluded from further analysis. Exclusion of these outliers did not affect the significance of the differences among groups.

**Table 1:** Gadolinium content in the nervous system

			**28 days post-treatment**	**56 days post-treatment**
	**Group**	**GBCA**		
**Cerebrum (nmol Gd/g)**				
SN rats	1	Gadoteridol	0.304 ± 0.088^b2,c3,c4^	0.132 ± 0.022^b2,c3,c4^
	*n*		8	8
	2	Gadobutrol	0.57 ± 0.12^b1,c4^	0.348 ± 0.097^b1,c4^
	*n*		8	7
	3	Gadoterate	0.554 ± 0.074^c1,c4^	0.321 ± 0.064^c1,c4^
	*n*		8	7
Healthy rats	4	Gadoteridol	0.087 ± 0.015^c1,c2,c3^	0.053 ± 0.007^c1,c2,c3^
	*n*		5	5
**Cerebellum (nmol Gd/g)**				
SN rats	1	Gadoteridol	0.328 ± 0.081^b2,b3^	0.264 ± 0.094^b4^
	*n*		8	8
	2	Gadobutrol	0.66 ± 0.23^b1,c4^	0.36 ± 0.10^b4^
	*n*		8	7
	3	Gadoterate	0.63 ± 0.13^b1,c4^	0.278 ± 0.036^c4^
	*n*		8	7
Healthy rats	4	Gadoteridol	0.16 ± 0.1^c2,c3^	0.084 ± 0.022^b1,b2,c3^
	n		5	5
**Peripheral nerves (nmol Gd/g)**				
SN rats	1	Gadoteridol	0.60 ± 0.29^c2,c3,a4^	0.166 ± 0.23^a3^
	*n*		8	7
	2	Gadobutrol	3.24 ± 0.81^c1,c4^	0.45 ± 0.22
	*n*		7	6
	3	Gadoterate	4.3 ± 1.4^c1,c4^	0.78 ± 0.58^a1,c4^
	*n*		8	8
Healthy rats	4	Gadoteridol	0.197 ± 0.035^a1,c2,c3^	0.114 ± 0.028^c3^
	*n*		5	5

Values presented as mean ± SD

^a^*p* < 0.05; ^b^*p* < 0.01; ^*c*^p < 0.001. The number after the letter of the significance level indicates the group* versus* which there is a difference. See main text for actual* p *values

**Table 2:** Gadolinium content in the blood, liver, femur, kidneys, and skin

			**28 days post-treatment**	**56 days post-treatment**
	**Group**	**GBCA**		
**Blood (nmol Gd/mL)**				
SN rats	1	Gadoteridol	0.101 ± 0.53	0.0116 ± 0.0046^a4^
	*n*		7	8
	2	Gadobutrol	0.099 ± 0.017^c4^	0.0167 ± 0.0086^b4^
	*n*		8	7
	3	Gadoterate	0.094 ± 0.026^b4^	0.0152 ± 0.072^a4^
	*n*		9	8
Healthy rats	4	Gadoteridol	0.045 ± 0.014^c2,b3^	0.004 ± 0.001^a1,b2,a3^
	*n*		5	4
**Liver (nmol Gd/g)**				
SN rats	1	Gadoteridol	2.2 ± 1.3^a4^	0.64 ± 0.28
	*n*		8	8
	2	Gadobutrol	4.2 ± 1.8^b4^	1.39 ± 0.81^a4^
	*n*		8	7
	3	Gadoterate	3.9 ± 2.1^b4^	2.0 ± 1.1^a4^
	*n*		9	7
Healthy rats	4	Gadoteridol	0.295 ± 0.087^a1,b2,b3^	0.132 ± 0.031^a2,a3^
	*n*		5	5
**Femur (nmol Gd/g)**				
SN rats	1	Gadoteridol	17.1 ± 5.4^b4^	15.4 ± 3.7^c4^
	*n*		8	8
	2	Gadobutrol	25.3 ± 9.5^b4^	24.9 ± 9.3^b4^
	*n*		8	7
	3	Gadoterate	20 ± 12^a4^	16 ± 10
	*n*		9	8
Healthy rats	4	Gadoteridol	6.6 ± 1.6^b1,b2,a3^	5.24 ± 0.19^b2,c1^
	*n*		5	5
**Kidneys (nmol Gd/g)**				
SN rats	1	Gadoteridol	206 ± 70^c2,b3,c4^	50 ± 36^b2,a3,a4^
	*n*		8	8
	2	Gadobutrol	991 ± 153^c1,c4^	265 ± 82^b1,b4^
	*n*		7	6
	3	Gadoterate	1286 ± 269^b1,c4^	283 ± 144^a1,a4^
	*n*		9	7
Healthy rats	4	Gadoteridol	9.0 ± 3.2^c1,c2,c3^	4.0 ± 1.7^a1,b2,a3^
	*n*		4	5
**Skin (nmol Gd/g)**				
SN rats	1	Gadoteridol	1.32 ± 0.38^c4^	0.475 ± 0.092^c4^
	*n*		8	7
	2	Gadobutrol	2.6 ± 1.6^b4^	0.71 ± 0.23^a4^
	*n*		8	6
	3	Gadoterate	2.0 ± 1.0^a4^	0.68 ± 0.43^a4^
	*n*		7	8
Healthy rats	4	Gadoteridol	0.42 ± 0.15^c1,b2.a3^	0.188 ± 0.005^c1, a2, a3^
	*n*		4	4

Values presented as mean ± SD. ^a^*p* < 0.05; ^b^*p* < 0.01; ^c^*p* < 0.001. The number after the letter of the significance level indicates the group *versus* which there is a difference. See main text for actual* p *values

Measurable amounts of Gd were detected in all tissue samples, except for some samples of blood and skin collected at day 56 after treatment. Extrapolated values were used for these samples when the amount of Gd was below the LLOQ.

As expected, Gd levels were higher in all tissues of SN rats than in healthy rats treated with gadoteridol, reaching statistical significance in almost all tissues for both post-treatment periods.

Gd content decreased in the post-treatment period between day 28 and day 56 for all GBCAs in all tissues except the femur, both in SN rats and healthy rats.

### Nervous system

The Gd concentrations in the cerebrum of SN animals at day 28 post-treatment were 0.304 ± 0.088 (mean ± SD), 0.57 ± 0.12, and 0.554 ± 0.074 nmol Gd/g for gadoteridol, gadobutrol, and gadoterate, respectively. At day 56 post-treatment, the Gd levels had fallen to 0.132 ± 0.022, 0.348 ± 0.097, and 0.321 ± 0.064 nmol Gd/g, respectively (Table 1 and Fig. 3). At both post-treatment timepoints, significantly (*p* ≤ 0.005 or *p* < 0.001) lower levels of Gd were found after administration of gadoteridol compared with gadobutrol or gadoterate while no significant (*p* ≥ 0.987) differences were noted between gadobutrol and gadoterate.

Similar findings were obtained for Gd levels in the cerebellum of SN animals. Lower Gd concentrations were noted at both day 28 post-treatment (0.328 ± 0.081, 0.66 ± 0.23, and 0.63 ± 0.13 nmol Gd/g for gadoteridol, gadobutrol, and gadoterate, respectively) and day 56 post-treatment (0.246 ± 0.094, 0.36 ± 0.10, and 0.278 ± 0.036 nmol Gd/g, respectively) although the differences were significant (*p* ≤ 0.003 or *p* < 0.001, respectively) only at day 28 post-treatment.

The mean Gd concentrations in peripheral nerves of SN animals were 0.60 ± 0.29, 3.24 ± 0.81, and 4.3 ± 1.4 nmol Gd/g for gadoteridol, gadobutrol, and gadoterate, respectively, at 28 days post-treatment, and 0.166 ± 0.023, 0.45 ± 0.22, and 0.78 ± 0.58 nmol Gd/g, respectively, at 56 days post-treatment. Significantly (*p* < 0.001) lower levels of Gd were found at day 28 post-treatment in the peripheral nerves of SN animals administered gadoteridol than in SN animals administered gadobutrol or gadoterate. Significantly (*p* ≤ 0.016) lower Gd levels were also noted at day 56 post-treatment *versus* gadoterate although the difference *versus* gadobutrol was not significant (*p* ≥ 0.122). No significant (*p* ≥ 0.420) differences were noted between gadobutrol and gadoterate.

### Non-nervous tissues

Significantly lower levels of Gd were found following gadoteridol administration than following administration of gadobutrol or gadoterate in the remaining portions of the kidneys of SN rats at both day 28 (*p* < 0.001, *p* ≤ 0.002) and day 56 (*p* ≤ 0.004, *p* ≤ 0.023) post-treatment (Table 2 and Fig. 3). The mean Gd concentrations were 206 ± 70, 991 ± 153, and 1283 ± 269 nmol Gd/g, for gadoteridol, gadobutrol, and gadoterate, respectively, at 28 days post-treatment, and 50 ± 36, 265 ± 82, and 283 ± 144 nmol Gd/g, respectively, at 56 days post-treatment.

Conversely, while lower levels of Gd were noted with gadoteridol in the liver, femur, and skin, none of the differences in SN animals were significant either at 28 days (*p* ≥ 0.053, *p* ≥ 0.271, *p* ≥ 0.244, respectively) or 56 days (*p* ≥ 0.107, *p* ≥ 0.165, *p* ≥ 0.24, respectively) post-treatment. No significant differences were seen in blood either at day 28 (*p* = 1.000) or day 56 (*p* ≥ 0.186) post-treatment.

## Discussion

To date, studies to evaluate Gd retention in rodent models of renal failure have looked at only linear GBCAs [20] or compared a solitary macrocyclic GBCA (gadoterate) with one [19] or two [21] linear GBCAs. Moreover, no studies have yet looked at Gd retention in renally compromised animals at multiple post-treatment timepoints.

Our study in SN rats (five sixths nephrectomy) determined Gd levels in nervous (cerebrum, cerebellum, peripheral nerves [lumbosacral plexus, sciatic, and tibial nerves]) and non-nervous (blood, liver, femur, kidney, skin) tissues at day 28 and day 56 after a total cumulative dose of 9.6 mmol/kg of each of three commercially available macrocyclic GBCAs gadoteridol, gadobutrol, and gadoterate. An additional comparison was made against a group of healthy (non-SN) male rats administered gadoteridol at a total cumulative dose of 9.6 mmol/kg using the same dosing schedule. Not unexpectedly, the levels of Gd in all tissues were higher in SN animals than in healthy animals at both post-treatment timepoints. This is consistent with a previous study in which renal insufficiency was shown to potentiate Gd retention in the brain (specifically, cortical brain, subcortical brain, cerebellar parenchyma, deep cerebellar nuclei, and brain stem) and bones of rats administered gadodiamide [20] and reflects the longer residence time of GBCAs in blood and body tissues due to markedly reduced renal elimination.

Among the SN animals, significantly lower levels of Gd were present in all nervous tissues and in the kidney following gadoteridol administration than following administration of gadobutrol or gadoterate at both post-treatment timepoints, apart from the cerebellum and peripheral nerves at day 56 after treatment. Lower levels of Gd following gadoteridol administration were also noted in all other non-nervous tissues at both post-treatment timepoints but the differences were not significant. Lower levels of Gd in brain and body tissues in the first weeks and months after gadoteridol administration compared with gadobutrol or gadoterate have been demonstrated consistently in healthy animals [15–18] and can be ascribed to the unique physico-chemical properties of the gadoteridol molecule that favour faster diffusion and clearance from brain interstitium and soft tissue organs (*i.e.*, low molecular weight and molecular features that minimise potential interaction with surrounding tissue matrix [31]). This study confirms that a similar Gd retention profile occurs also in rats with severe renal impairment.

Recently, Alkhunizi et al. [32] reported significantly higher levels of retained Gd in the spinal cord and particularly the peripheral nerves of rats given intraperitoneal injections (0.6 or 2.5 mmol/kg) of gadodiamide compared to gadoterate and noted that while neither GBCA affected spatial working memory or hippocampal cell proliferation and maturation, gadodiamide but not gadoterate led to significant (*p* < 0.001) pain hypersensitivity to thermal and mechanical stimuli. Our study is the first to include a determination of Gd levels in peripheral nerves of rats after intravenous administration of GBCAs. Previous studies have evaluated Gd retention in peripheral nerves only after intraperitoneal administration in rats [32] or after intravenous administration in mice [14, 33]. In common with other tissues evaluated, greater levels of retained Gd were noted with gadobutrol and gadoterate than with gadoteridol: at day 28 after treatment, the Gd content in peripheral nerves of SN rats treated with gadobutrol or gadoterate was 5.4 and 7.2 times higher, respectively, than in SN rats treated with gadoteridol. Considering that collagen is the main component of the extracellular matrix of peripheral nerves (comprising about 90% type I collagen [34, 35]), our findings are consistent with those of a recent *in vitro* study, which showed that gadoteridol accumulates to a lower degree in collagen than both gadoterate and gadobutrol [36]. Our study also demonstrated that Gd content was higher in peripheral nerves than in both the cerebrum and cerebellum, particularly at day 28 after treatment. This observation may reflect the fact that the peripheral nervous system contains substantial amounts of collagen which plays a role in nerve function, while collagen is present only in the meninges and vasculature of the central nervous system [37]. This, together with the absence of the blood–brain barrier in the peripheral nervous system, may explain why Gd levels were higher in peripheral nerves both in SN and healthy rats. In this regard, a twofold higher Gd content was determined in peripheral nerves compared with the cerebrum both in SN and healthy rats following gadoteridol administration.

We determined Gd levels at two post-treatment timepoints. The first (day 28) was selected because it has frequently been used in previous studies [7, 14–16] and the second (day 56) to permit monitoring of possible differences in Gd elimination within a timeframe in which Gd content is reliably measurable. Several studies have shown that differences between GBCAs tend to disappear at later timepoints than those investigated in our study (*e.g.*, day 180 and day 360) and that Gd levels at these later timepoints tend to approach or supersede the LLOQ values [17, 38, 39]. These later timepoints, however, are of limited relevance given that 1 rat year corresponds to approximately 30 human years [40]. It is the possible effects of retained Gd within the first weeks and months that are of particular importance when the Gd levels are necessarily higher. Lower levels of Gd were determined at day 56 after treatment compared to day 28 after treatment in all tissues of both SN and healthy rats, although the differences were much less pronounced in the femur, as noted elsewhere [14, 19]. This is likely due to the specific nature of the bone matrix, where diffusion of GBCAs is different from that in other soft tissues.

It is widely accepted that the overall levels of retained Gd after the administration of macrocyclic GBCAs are very low and that no detrimental effects on human health have been determined. Nevertheless, it is worth taking into consideration of the differences among the levels of retention observed in animals following the administration of the available GBCAs, particularly when dealing with critical patients with minimal or no renal function or when several administrations of GBCA are needed.

A limitation of our study is that, whereas the adopted nephrectomy model simulates the loss of functioning nephrons seen in patients with chronic kidney disease, it does not reproduce the progressive alterations that are typically found in chronic kidney disease and, therefore, our study cannot mimic the impact of these alterations on GBCA clearance. Nevertheless, our findings provide further evidence that there are differences between macrocyclic GBCAs, which should be taken into consideration when selecting a GBCA for clinical use. A second limitation is that we only compared the three macrocyclic GBCAs that were commercially available at the time of the study. A fourth macrocyclic GBCA, gadopiclenol (Vueway/Elucirem), was recently approved by the US Food & Drug Administration and may offer an alternative to the three GBCAs described here. Initial studies have shown similarly low levels of Gd retention after gadopiclenol administration, both in healthy rats and in rats with severe renal impairment [30, 41].

In conclusion, lower Gd levels were observed following repeated intravenous exposure of SN rats to gadoteridol at a cumulative dose of 9.6 mmol/kg than following exposure to equivalent doses of gadobutrol and gadoterate. Differences were apparent in nearly all sampled tissues at both day 28 and day 56 after treatment. In the femur, the differences among GBCAs were not significant, and the decrease in Gd levels between day 28 and day 56 was less apparent.

## Data Availability

The datasets used and/or analysed during the current study are available from the corresponding author on reasonable request.

## Abbreviations

ANOVA: Analysis of variance
GBCA: Gadolinium-based contrast agent
ICP-MS: Inductively coupled plasma/mass spectrometry
LLOQ: Lower limit of quantitation
NSF: Nephrogenic systemic fibrosis
SD: Standard deviation
SN: Subtotally nephrectomised
